# A clinical decision support system for the diagnosis of probable migraine and probable tension-type headache based on case-based reasoning

**DOI:** 10.1186/s10194-015-0512-x

**Published:** 2015-04-01

**Authors:** Ziming Yin, Zhao Dong, Xudong Lu, Shengyuan Yu, Xiaoyan Chen, Huilong Duan

**Affiliations:** College of Biomedical Engineering and Instrument Science, Zhejiang University, Zheda Road 38, Hangzhou, Zhejiang 310008 China; International Headache Center, Department of Neurology, Chinese PLA General Hospital, Beijing, 100853 China

**Keywords:** Headache, Clinical decision support, Case-based reasoning

## Abstract

**Background:**

The overlap between probable migraine (PM) and probable tension-type headache (PTTH) often confuses physicians in clinical practice. Although clinical decision support systems (CDSSs) have been proven to be helpful in the diagnosis of primary headaches, the existing guideline-based headache disorder CDSSs do not perform adequately due to this overlapping issue. Thus, in this study, a CDSS based on case-based reasoning (CBR) was developed in order to solve this problem.

**Methods:**

First, a case library consisting of 676 clinical cases, 56.95% of which had been diagnosed with PM and 43.05% of which had been diagnosed with PTTH, was constructed, screened by a three-member panel, and weighted by engineers. Next, the resulting case library was used to diagnose current cases based on their similarities to the previous cases. The test dataset was composed of an additional 222 historical cases, 76.1% of which had been diagnosed with PM and 23.9% of which had been diagnosed with PTTH. The cases that comprised the case library as well as the test dataset were actual clinical cases obtained from the International Headache Center in Chinese PLA General Hospital.

**Results:**

The results indicated that the PM and PTTH recall rates were equal to 97.02% and 77.78%, which were 34.31% and 16.91% higher than that of the guideline-based CDSS, respectively. Furthermore, the PM and PTTH precision rates were equal to 93.14% and 89.36%, which were7.09% and 15.68% higher than that of the guideline-based CDSS, respectively. Comparing CBR CDSS and guideline-based CDSS, the p-value of PM diagnoses was equal to 0.019, while that of PTTH diagnoses was equal to 0.002, which indicated that there was a significant difference between the two approaches.

**Conclusions:**

The experimental results indicated that the CBR CDSS developed in this study diagnosed PM and PTTH with a high degree of accuracy and performed better than the guideline-based CDSS. This system could be used as a diagnostic tool to assist general practitioners in distinguishing PM from PTTH.

## Background

The International Classification of Headache Disorders (ICHD) published by the International Headache Society (IHS) has been proven to be effective and has been widely applied to clinical practice worldwide [1,2]. However, practitioners are often confronted with patients whose symptoms meet some but not all diagnostic criteria. In some studies, researchers have found that some specific types of headaches share multiple similarities [3-6]. For example, many migraine attacks are accompanied by tension headache-like symptoms, such as neck pain. In addition, tension-type headaches are often accompanied by migraine headache-like symptoms, such as photophobia, phonophobia, and aggravation by routine physical activity. Among these overlaps, the overlap between probable migraine (PM) and probable tension-type headache (PTTH) is most common due to their high individual incidence rates [7-10]. Since PM and PTTH have different preventative therapies, the differential diagnosis of these two types of headaches is necessary.

The development of clinical decision support systems (CDSSs) for the diagnosis of primary headaches has long been a major research topic. Most existing headache CDSSs are based on ICHD criteria [11-14]. However, research has shown that none of these CDSSs are capable of differentiating among primary headaches with overlapping features. In our previous studies [15,16], the guideline-based CDSS did not perform adequately when faced with PM and PTTH. Thus, in this study, a feasible computer-aided diagnosis method for differentiating between these two types of headaches was proposed.

In clinical practice, headache experts diagnose these headaches based on their clinical experience by recalling key indicators and previously solved cases. In an attempt to emulate this expertise and reasoning, a CDSS based on case matching and recommendations, or case-based reasoning (CBR), was developed. CBR, an artificial intelligence technique, is the process of solving new problems based on the solutions of similar, previously solved problems, and is considered to be one of the most effective methods of managing implicit knowledge, such as intuition and experiences. In CBR, in order to solve a new problem, cases with features that are most similar to the new problem are retrieved from a case library, and their solutions are considered for reuse. This method is very suitable for the diagnosis of PM and PTTH. CBR has been applied to many other medical areas as well. For example, Koton [17] developed a case-based system named CASEY for the diagnosis of heart complications, Guessoum et al. [18] presented a decision-based support system for the diagnosis of chronic obstructive pulmonary disease, and Sharaf-El-Deen et al. [19] proposed a hybrid case-based reasoning approach for the diagnosis of breast cancer and thyroid diseases. All of these systems have been successful in local settings. In this study, a CBR CDSS was developed in order to assist general practitioners in differentiating between PM and PTTH.

## Methods

### Ethics

The protocol used in this study was approved by the Chinese PLA General Hospital ethics committee in Beijing, China.

### Overview

**Figure 1:**
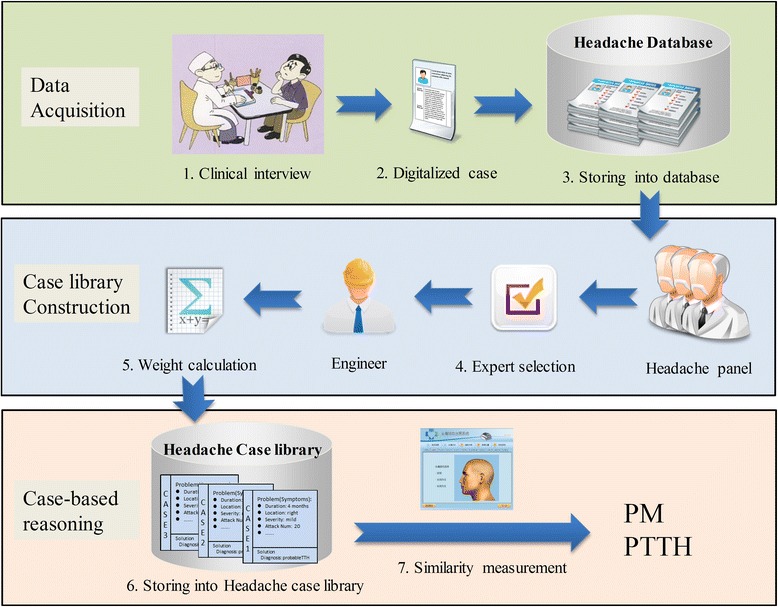
**The whole process of case library construction and recommendation.**

As shown in Figure 1, the CBR method proposed in this study was composed of three main steps: data acquisition, case library construction, and case-based reasoning. Each step was further divided into a total of seven sub-steps.

### Clinical data acquisition

A CBR system must contain a library of previously solved cases obtained from routine clinical practice. Physicians acquire complete medical histories of headache patients through clinical interviews. The data sheet for acquiring data using in the clinical interview is shown in Table 1. The options of some items in Table 1 are listed in Table 2, such as location of pain et al. The patients’ symptoms are recorded in a digitalized format and stored into the headache database. The researchers from the International Headache Center collected numerous headache cases. These cases were used as the data source in our headache database. Since the headache case library used in this study was a subset of the headache database, the individual cases were screened by a three-member panel before being stored in the library. This process is described in the next section.

**Table 1:** **The data sheet using in the clinical interview**

**Item**	**Example**
Gender	Female
Age	43
unilateral	Yes
bilateral	No
Pain quality	Pulsating
Pain intensity	10
Date of headache onset	5/17/2014
Duration of pain episodes	24 hours
Attack frequency	20/month
Attack with fixed period	No
The number of attacks	50
Aggravation by or causing avoidance of routine physical activity	Yes
Persistent headache, daily from its onset	No
Years of smoking	22 years
Years of drinking	0 year
How many cups of tea per day	1
How many cups of coffee per day	0
Family medical history	Yes
Location of pain*	Crown, Tempus
Precipitating factors*	Menstruation
Relieving factors*	Stay in dark room
Accompanying symptoms*	Photophoby, Phonophobia
Aura*	None
Premonitory symptom*	Feel weak

*the options of these items are shown in Table 2.

**Table 2:** **The options of some items in Table** 1

**Item**	**Options**
Location of pain	Tempus | Crown | Forehead | Pars orbitalis | Face | Ear | Occiput | Neck
Precipitating factors	Sleeplessness | Mood swings | Food | Activities | Weather change | Menstruation | Hard light | Smell | Noise
Relieving factors	Lay Down | Stay in Dark Room | Massage | Hot Compress | Cold Compress | Fast Walk | Exercise | Stand | Pregnancy
Accompanying symptoms	Nausea | Vomit | Photophoby | Phonophobia
Aura	Visual | Sensory | Speech and/or language | Motor | Brainstem | Retinal
Premonitory symptom	Loquacity | Depression | Irritability | Dysesthesia | Stiff neck | Thirstiness | Yawn | Drowsiness | Fidget | Poor appetite | Photophobia | Phonophobia | Constipation | Attention Disorder | Diarrhea | Diuresis | Dysphasia | Feel weak | Feel dizzy | Feel cold

### Case library construction

A three-member panel preprocessed the cases used in the headache database. Only cases with a PM or PTTH diagnosis were retained; any cases that contained incomplete or inaccurate information were excluded. Furthermore, the remaining cases were processed in order to ensure that the diagnosis was correct. Next, engineers calculated the weight of each feature, or symptom, in each case. The resulting filtered headache case library consisted of 676 cases, 56.95% of which had been diagnosed with PM and 43.05% of which had been diagnosed with PTTH.

#### Patient feature selection

An appropriate case structure increases the success rate of case matching. In this study, each headache case consisted of two components: the symptoms and diagnosis. The diagnosis was considered the solution of the case. The symptoms consisted of 74 different headache symptoms selected by the three-member panel that were used to represent the features of each headache case. These features not only included the ICHD-3 beta diagnostic indicators, such as headache duration, headache intensity, nausea, vomiting, photophobia, and phonophobia, but also the precipitating factors, relieving factors, premonitory symptoms, and family medical history. A sample of the structured headache case library is shown in Table 3.

**Table 3:** **Sample headache case library**

**Case**	**Duration**	**Location**	**Intensity**	**Frequency (/m)**	**nausea**	**vomiting**	**Menstruation**	**Diagnosis**
1	24	Tempus	8	5	True	False	Yes	PM
2	24	Forehead	9	7	True	True	Yes	PM
3	48	Crown	5	13	False	False	No	PM
4	2	Tempus	5	7	False	False	No	PTTH
5	120	Tempus	7	1	False	False	Yes	PTTH
6	24	Tempus	6	8	False	False	Yes	PM

#### Weight calculation

The weight of each feature was used to represent the importance of that feature in measuring similarity. The higher the weight of an attribute, the more relevant it was considered to be. Thus, establishing accurate weights was of high importance. In this study, the weights of the attributes were calculated using an evolutionary approach: the Genetic Algorithm (GA). The GA is a search heuristic that mimics the process of natural evolution. This heuristic is routinely used to generate practical solutions to optimization and searching problems using techniques inspired by natural evolution, such as inheritance, mutation, selection, and crossover. First, an equal weight was assigned to each feature. Then, the GA searched the solution space and determined the optimized weight of each feature. The weights were stored in the case library and utilized as the case similarity measurement parameters.

### Case-based reasoning

**Figure 2:**
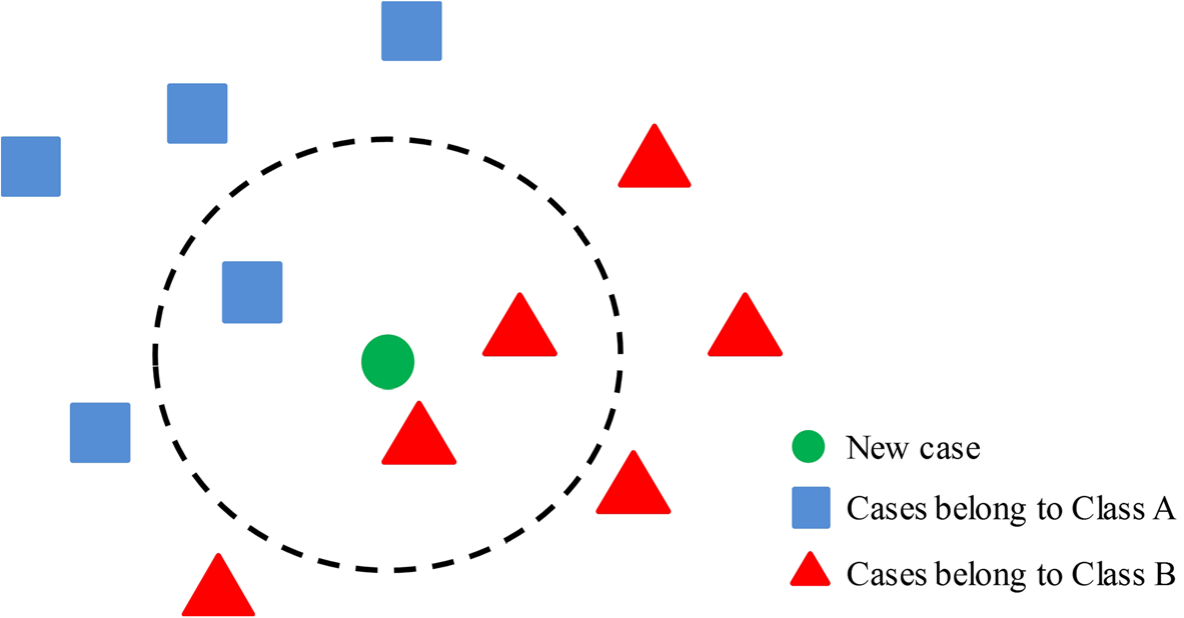
**The principle of case similarity measure.**

The proposed CBR CDSS could provide recommendations to physicians when unsure of a patient’s diagnosis. These recommendations are based on similarity measurements between new cases and previous cases in the case library. Although many methods that measure the similarity between two cases exist, the K-Nearest Neighbor (KNN) method was implemented due to its relative simplicity and higher accuracy than other more complicated algorithms. In the KNN method, the features are first compared in order to determine the similarity of between a new case and previous cases. Next, the distance between the new case and each case in the case library is calculated according to a predefined formula. Then, K cases with minimal distances are selected as the neighbors of the new case; the new case is classified according to its neighbors and assigned to the class most common among its K nearest neighbors, as shown in Figure 2. If K = 1, the nearest case is regarded as the solution. In this study, an equation, which was presented in [20], was adopted to measure the similarity between a new case and all previously solved cases in a case library. The equation is given as

1 $$ \mathrm{similarity}\left(\mathrm{A},\mathrm{B}\right)={\displaystyle {\sum}_{i=0}^nf\left({A}_i,{B}_i\right)\times {w}_i} $$

where A is the problem case, B is an existing case in the case library, n is the number of attributes/features of each case, i is the individual features of each case from 0 to n, f is the similarity function of feature i in cases A and B, and w is the weight of each individual feature based on the number of matches, which is calculated in the last step.

Note that the recommended results obtained from the system cannot be automatically denoted the final diagnostic result. The results must be confirmed by a physician, who will determine whether the recommended K cases with the highest similarities meet the clinical needs of a patient.

### System description

The core functionality of this system was comprised of structured symptomatic input, computer-aided diagnosis, and automated report generation. Structured symptomatic input generalizes digitalized forms of cases and further analyzes cases. The CBR technique performs computer-aided diagnosis as the system ranks similar cases based on their similarity scores. Automated report generation reduces the time consumed by writing reports and increases work efficiency.

The proposed system, a web-based system, was deployed on a cloud platform in this study. It not only acted as a complete computer-aided diagnostic system, but also as a data center for primary headaches. A profile that could be updated by a physician at each successive follow-up appointment was created for each patient. A three-member panel checked each case in order to ensure accurate diagnoses and select representative cases for storage in the case library at regular intervals.

### Validation

#### Test data set

The test data set was comprised of 222 previous cases that were collected by the International Headache Center in Chinese PLA General Hospital. These data were not included in the CBR CDSS case library. The diagnosis of each case was determined by a three-member panel as either PM or PTTH; 76.1% of the cases were PM.

#### Evaluation metrics and method

In this study, the effectiveness of the proposed system was assessed using the recall rate, precision rate, F-score, and accuracy. The recall rate is a measure of completeness, and the precision rate is a measure of exactness. The F-score is the harmonic mean of the recall rate and precision rate. It gives equal weight to the precision and recall rates. The accuracy of the system is the percentage of the test data that were correctly classified by the classifier. The formulas for each of these evaluation measures are as follows:

2 $$ \mathrm{Recall} = \frac{TP}{TP+FN} $$

3 $$ \mathrm{Precision} = \frac{TP}{TP+FP} $$

4 $$ \mathrm{F}=\frac{2\times precision\times recall}{precision+ recall} $$

5 $$ \mathrm{accuracy} = \frac{TP+TN}{N} $$

where TP, FP, FN, and TN refer to the number of true positives, false positives, false negatives, and true negatives, respectively, and N refers to the number of test data.

In addition to the above metrics, the Receiver Operator Characteristic (ROC) curve is another useful visual tool used to compare the accuracies of different algorithms. The ROC is a graphical plot of the sensitivity (the true positive rate) versus the false positive rate (one minus the true negative rate) of a binary classifier system as its discrimination threshold is varies. When the performance of a method is reflected by the area under its ROC curve (AUC), its effectiveness becomes apparent. As the area under the curve increases, the performance of the classifier also increases.

#### Experimental design

Several evaluations were designed in order to verify the effectiveness of the proposed system by answering three main questions: 1) To what value should parameter K be set in order to ensure that the proposed approach would achieve its optimal accuracy? 2) Did the proposed weighting operation improve the diagnostic performance of the system? 3) Did the CBR CDSS more aptly diagnosis PM and PTTH compared to the guideline-based CDSS?

1. Experiment 1: the variation in performance as the value of K varies
  In the KNN method, the value of K is most influential to the recommendation performance. An appropriate K value could improve the accuracy of the recommendation. In this experiment, the value of K was increased from 1 to 51, and the accuracy of each value of K was recorded.
2. Experiment 2: the comparison between the weighted CBR and unweighted CBR
  Since there is no weighting operation in traditional CBR, each feature is assigned a weight of 1. Assigning different weights to the features of each case was expected to improve the recommendation accuracy. In order to validate this, the recommendation accuracies of the weighted CBR and unweighted CBR were compared using AUC and Pearson’s chi-square test. A p-value of 0.05 or less was used to indicate statistical significance. SPSS software for Windows (Version 18.0) was used for the statistical analyses.
3. Experiment 3: the comparison between the CBR CDSS and the guideline-based CDSS
  The PM and PTTH diagnostic performances of the guideline-based CDSS and CBR CDSS were compared. A statistical analysis was also performed using Pearson’s chi-square test in order to determine whether a significant difference existed between these two methods. A p-value of 0.05 or less was used to indicate statistical significance.

## Results

### Weight

Weight was used to represent the relative importance of each of the features during the decision-making process. Table 4 shows the top 20 features and their weights.

**Table 4:** **The top 20 features and their weights**

**Feature**	**Weight**	**Feature**	**Weight**
Date of headache onset	1	Nausea	.954
Aggravation by routine physical activity	1	Cold compresses	.949
Location of pain	1	Photophobia	.946
Gender	1	Attack with fixed period	.929
Duration of pain episodes	1	Family medical history	.896
Pain quality	1	Menstruation related factor	.875
Vomiting	.994	Pain intensity	.874
The number of attacks	.96	Exercise	.864
Phonophobia	.957	Attack frequency per month	.839

During the weight calculations, most of the weights were determined to be consistent with the experience of the specialists’. For example, nausea is considered to be an important diagnostic feature for differentiating migraine and tension-type headaches [21]. Thus, as shown in Table 4, nausea and vomiting were assigned higher weights. In addition, some features that are not included in the ICHD-3 beta criteria, such as family history, exercise, and menstruation-related factors, were also given higher weights.

### Experiment 1

**Figure 3:**
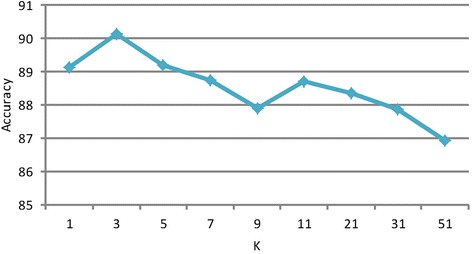
**The changes of accuracy with K increasing.**

The setting of a parameter K significantly impacts recommendation accuracy. Since K is the number of voting neighbors and the KNN method uses a majority vote scheme, odd-value samples ranging from 1 to 51 were studied. Although it is often assumed that better accuracy values can be obtained if the number of neighbors is increased, this is not the case. As shown in Figure 3, the maximum accuracy was achieved when the value of K was equal to 3. Thus, when K was more than 3, the accuracy decreased as K increased. Therefore, the value of K was set at 3.

### Experiment 2

The ROC curves of the weighted CBR and unweighted CBR were compared, and the AUC of the weighted CBR (0.983) was determined to be significantly larger than that of the unweighted CBR (0.799). This illustrated not only that the weighting operation was necessary for case matching, but also that the proposed approach was superior to the unweighted CBR approach. A statistical analysis between the weighted CBR and unweighted CBR was performed using the Pearson’s chi-square test. The PM chi-square value was equal to 9.571, and the p-value was 0.002 < 0.05. Likewise, the PTTH chi-square value was equal to 18.002, and the p-value was 0 < 0.05. The results indicated that there was a significant difference between the two approaches. Thus, the weighting operation was necessary for CBR.

### Experiment 3

Table 5 shows the recall rate, precision rate, and F-score of the CBR CDSS and the guideline-based CDSS for PM and PTTH diagnosis. As shown in this table, the performance of the CBR CDSS was better than that of the guideline-based CDSS. Moreover, the Pearson’s chi-square test was used to compare the CBR CDSS and the guideline-based CDSS. The p-values for both the PM and PTTH were less than 0.05, and the null hypothesis was rejected in favor of the alternative hypothesis (a significant difference exists between the two methods).

**Table 5:** **The diagnostic performance for two systems**

	**CBR CDSS**			**Guideline-based CDSS**		
	**Recall (%)**	**Precision (%)**	**F-score (%)**	**Recall (%)**	**Precision (%)**	**F-score (%)**
PM	97.02	93.14	95.04	62.71	86.05	72.55
PTTH	77.78	89.36	83.17	60.87	73.68	66.67

**Figure 4:**
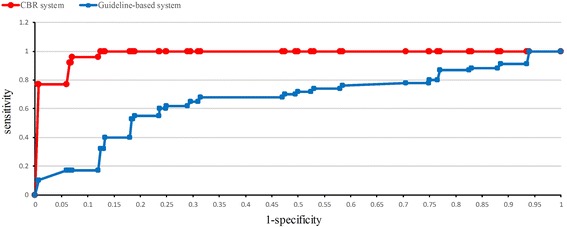
**The ROC curve of CBR system and guideline-based system.**

The same conclusion was found in the ROC curves as well. Figure 4 shows the ROC curves of the two methods. The AUC of the CBR CDSS (0.983) was larger than that of the guideline-based CDSS (0.661). The closer the area was to one, the more accurate the corresponding approach was determined to be. The performance of the CBR CDSS was obviously better.

## Discussion

In this study, a CDSS for the diagnosis of headaches was developed using a CBR technique. The system demonstrated a higher diagnostic performance for PM and PTTH than that of a guideline-based CDSS. The proposed CDSS exhibited multiple advantages. Since CBR is a data-based reasoning approach, it is particularly effective in fields where summarizing explicit knowledge is difficult; thus, the proposed CDSS prevented the bottleneck of knowledge acquisition. In addition, unlike other machine learning methods, CBR does not require a model; instead, it calculates the similarities between the new and previous cases and, thereby, conserves time and effort. Furthermore, the proposed system gained intelligence as the number of cases increased due to the increased probability of a case with a high similarity.

The system was designed to assist with distinguishing PM from PTTH. The following example was used to illustrate the effectiveness of this system in practice. Patient A, a 24-year-old female, had headaches for four years that lasted approximately two days and were unilateral, pulsating, mild in intensity, and aggravated by routine physical activity with no nausea, vomiting, photophobia, or phonophobia. In addition, Patient A’s headache attacks were obviously related to her menstrual cycles, and her mother and sisters had experienced similar headaches. Determining the type of headache Patient A has is difficult since the diagnostic criteria of both PM and PTTH are met according to ICHD-3 beta. Based on this evidence, a similarity measurement of this case to previous clinical cases was performed. One case retrieved from the library, a 49-year-old female, had experienced recurrent attacks for more than 30 years; each attack lasted half a day or more. The patient’s headaches were unilateral, pulsating, severe in intensity, and aggravated by routine physical activity, with no nausea, vomiting, photophobia, or phonophobia. Furthermore, her attacks often occurred during menstruation. The similarity measurement of these two cases was equal to 0.965. Based on the information provided by the retrieved case, Patient A was determined to likely be experiencing migraine headaches.

**Figure 5:**
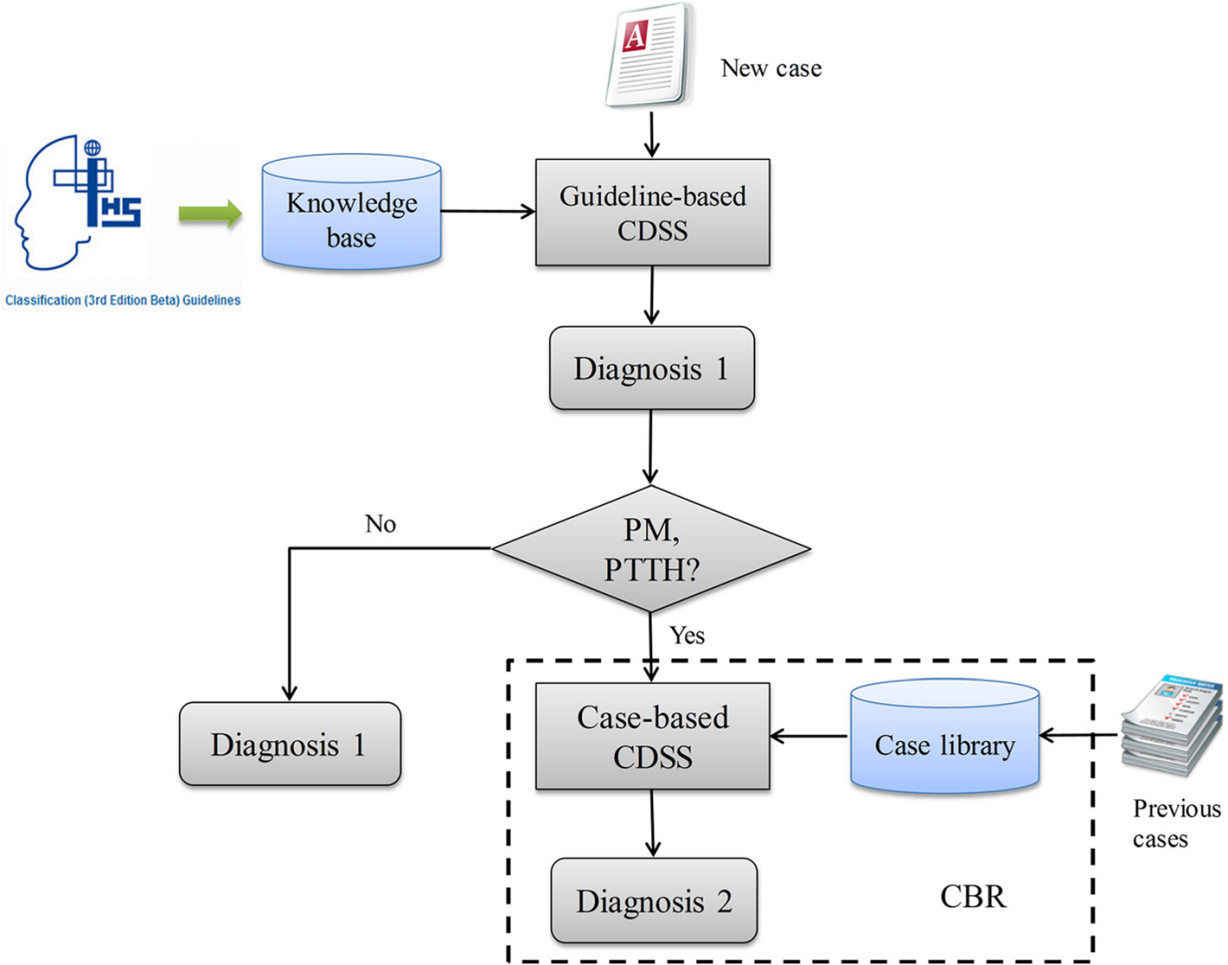
**The collaboration mechanism of the guideline-based CDSS and the CBR CDSS.**

In a previous study, a guideline-based clinical decision support system (CDSS) for the diagnosis of primary headaches was developed based on the latest ICHD-3 beta and was validated in a prospective study at the International Headache Center in Chinese PLA General Hospital [16]. The results revealed a high degree of accuracy in recognizing most types of primary headaches; however, it was unable to accurately diagnose PM and PTTH when symptoms overlapped. The CBR CDSS presented in this study could be considered a complementary system to the previously developed guideline-based CDSS. The collaborative mechanisms of these two systems are shown in Figure 5. The system first evaluates a new case based on the guideline-based CDSS first and then makes the first diagnosis (Diagnosis 1). Then, the system determines whether Diagnosis 1 is PM or PTTH; if not, the first diagnosis is directly recommended to physicians. Otherwise, CBR is used to evaluate the case for further confirmation and a second diagnosis (Diagnosis 2). With the combination of these two systems, most primary headaches could possibly be diagnosed with a high degree of accuracy. The system as a whole could be implemented in primary care units and community hospitals in order to assist general practitioners and primary care physicians in improving the diagnosis of primary headaches.

However, the proposed CBR CDSS has some limitations. For example, the number of cases in the case library is inadequate due to the complexity of headaches. In addition, since some complicated diseases were not included in the case library, the diagnostic accuracy is not as high as it could be. Although this problem would be solved with the addition of new cases into the case library, the computing time would grow significantly as a result since CBR measures the distance between a new case and each case in the case library. The recent development of techniques used to manage large amounts of data could be a viable solution to this problem.

## Conclusions

In this study, a weighted CBR method was applied to CDSSs in order to develop a CBR CDSS capable of differentiating between two types of probable primary headaches (PM and PTTH), which often confuse physicians in clinical practice. The results of the experiments indicated a high degree of accuracy in recognizing these two types of headaches and a dramatic improvement compared to guideline-based CDSSs. The accurate diagnosis of these types of headaches is imperative since their treatments involve different preventative therapies.

In future studies, the proposed CBR CDSS will be further evaluated in order to determine its validity. A prospective study and multicenter validation will be performed before it is recommended for routine clinical use. The final aim of this study is the development of a reliable CDSS for the diagnosis of different types of headaches using multiple techniques in order to assist physicians in primary care units and community hospitals and thereby improve the diagnosis and treatment of headaches.

### Consent

Written informed consent was obtained from the patient for the publication of this report and any accompanying images.
